# Diagnosis of hydatidiform moles using *p57 immunohistochemistry* and *chromogenic insitu hybridization*: A retrospective study

**DOI:** 10.18502/ijrm.v22i9.17478

**Published:** 2024-11-14

**Authors:** Mojgan Akbarzadeh-Jahromi, Tara Taheri, Fatemeh Sari Aslani, Akbar Safaei, Fatemeh Pouraminaee, Marjan Zare

**Affiliations:** ^1^Pathology Department, School of Medicine, Shiraz University of Medical Sciences, Shiraz, Iran.; ^2^Maternal-Fetal Medicine Research Center, Shiraz University of Medical Sciences, Shiraz, Iran.

**Keywords:** Hydatidiform mole, CISH protein, Human, Pathology, Clinical.

## Abstract

**Background:**

Chromogenic in situ hybridization(*CISH*) and immunohistochemistry analysis for *p57* are ancillary studies discriminating partial hydatidiform mole (PHM), complete hydatidiform mole (CHM), and non-molar hydropic abortion (HA).

**Objective:**

It aimed to study *CISH* with a probe to chromosome 17(*CISH17*) and chromosome 2(*CISH2*) discriminating chromosomal ploidy of PHM, CHM, and HA; in addition, their surrogacy value in the evaluation of triploid and diploid in product of conception specimens (POCs) was evaluated.

**Materials and Methods:**

44 statistically significant POCs were selected retrospectively. The Kappa agreement coefficients, sensitivity, specificity, and accuracy with 95% confidence interval (95% CI) were reported.

**Results:**

PHM, CHM, and HA were diagnosed to be 23, 17, and 3 cases based on both *CISH2* and *CISH17* resulting in their complete discrimination between PHM and HA (23 vs. 3). The Kappa agreement coefficient was 95.4% (p < 0.001) when diagnosing the PHM (23), CHM (20), and HA (1). In addition, the accuracy, sensitivity, and specificity were 95.26% (95% CI: 84.25–99.38), 100% (95% CI: 85.18–100), and 95% (95% CI: 76.18–99.88), respectively. The power analysis on *CISH2* and *CISH17* tests discriminating between triploid and diploid in POCs was estimated to be 100%.

**Conclusion:**

Based on the current finding, *CISH2* and *CISH17* enjoyed perfect agreement in diagnosing chromosomal ploidy; in addition, their absolute power discriminating between triploid and diploid revealed that they could be used as surrogate markers for ploidy. Prospective studies on fresh specimens are suggested comparing the *CISH* method's accuracy with flow cytometry karyotyping and fluorescence in situ hybridization.

## 1. Introduction

Spontaneous abortion is pregnancy loss before the 20 ${\;}^{\text{th}}$ wk of gestational age, which occurred in 10–15% of pregnancies (1–3). In spontaneous abortions, the distinction between hydatidiform moles (HMs) including complete hydatidiform mole (CHM) and partial hydatidiform mole (PHM) and nonmolar hydropic abortion (HA) are essential (1, 4–8). HMs cannot be diagnosed by morphologic examination alone; DNA ploidy analysis is useful in the classification of PHMs as triploids and CHMs as diploids. Loss of *p57* immunohistochemistry, which is a protein product of the paternally imprinted and maternally expressed Cyclin Dependent Kinase Inhibitor 1C gene, occurs in all CHMs but not in PHMs and is used to differentiate between PHM and CHM. Persistence or transformation into a malignant disease that requires chemotherapy has been estimated to occur in 0.5–5% of PHMs (9, 10); hence, an accurate diagnosis of PHM is essential (10, 11). Also, the distinction between trisomic pregnancy and PHM is difficult in early moles (9, 10, 12, 13). The PHM diagnosis is done with ploidy study;flow cytometry karyotyping is a versatile technique needing freshly frozen specimens; in addition, fluorescence in situ hybridization (*FISH*) is another effective DNA ploidy with accuracy in both freshly frozen and paraffin-embedded specimens (10, 14). However, *FISH* requires immunofluorescence staining technique which makes it costly and is not accessible in most laboratories. Since fresh tissue samples for cytogenetics are not always available and many laboratories do not perform ploidy analysis on paraffin sections (9, 15), we studied chromogenic in situ hybridization (*CISH*) with a probe to the chromosome 17 (*HER 2*) gene (*ZytoDot HER-2/CEN-17*)(*CISH17*) and chromosome 2 (*CISH2*) discriminating chromosomal ploidy of PHM, CHM, and non-molar HA ancillary with *p57* immunohistochemistry; formalin-fixed, paraffin-embedded tissue samples were readily available in laboratory archives and have a quick turnaround time. We postulated that they could also be used as a surrogate marker for ploidy in for the evaluation of triploid and diploid in product of conception specimens (POCs).

## 2. Materials and Methods

### Study design

**Figure 1:**
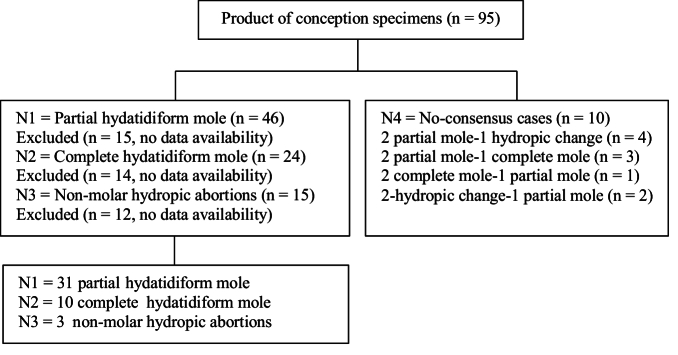
Case selection flowchart.

In a retrospective study, all 95 POCs having either CHM, PHM, or HA referred to Shahid Faghihi and Hazrate Zeinab hospital histopathological centers in Shiraz, Iran. Between June 2010–2015, 44 statistically significant POCs were selected based on the morphological characteristics agreed upon by 3 pathologists. Maternal and gestational ages ranged between 18 and 40 yr and 8–25 wk, respectively. The case selection flowchart is presented in figure 1.

### Sample size consideration

We, retrospectively, included all statistically significant POCs diagnosed in histopathological centers over a 5-yr period.

### Morphological review

Trophoblast Atypia (T. Atypia) was described as triple variation in nuclear size, nuclear widening with pleomorphism, and/or hyperchromasia. T. Atypia was detectable at standard magnification ( × 100). It was measured using syncytiotrophoblast, intermediate trophoblast, cytotrophoblast, and areas. Cistern formation (Cistern) was described as completely “acellular cavities having edema fluid within the center of the villi”, which involved a minimum 50% of the terminal villi. Though the villi offered deteriorating alterations, well-formed cisterns were considered. “Pseudo-cisterns subsequent bends in the stem villi or in the placental membranes” were omitted. Multifocal trophoblast proliferation (MTP) needs the manifestation of 2 or more distinct foci of trophoblast proliferation distributed along the surface of the villi. Although circumferential trophoblast was seldom identified, it was also included. Lace-like was defined as prominent intracytoplasmic lacunae developing in intermediate trophoblast and cytotrophoblast, usually in an extravillous distribution and distinguishable at × 40 to × 100 magnification. Large trophoblast inclusions (large-INC) resulted from dividing across irregular villous outlines, which resulted in irregularly shaped inclusions of various sizes but > 0.2 mm in diameter. Scalloping was described by several regular invaginations in the contours of enlarged villi. Small trophoblastic inclusions (small-INC) that did not appear in continuity with the surface trophoblast were rounded no more than 0.2 mm in diameter. Fibrillary collagen (fibrillary.C) included curvy collagen bundles leaning along the long axis of the larger villi.

All specimens were independently reviewed by 3 pathologists and classified as PHM, CHM, and HA based on morphology findings of table I (1, 16).

### p57 Immunohistochemistry

Sections of 4 µm tissue were located on covered slides and immune stained using a “Leica Bond Max Autostainer (Leica Biosystems, Wetzlar, Germany), lyophilized mouse monoclonal antibody (clone 25B2 at 1:50 dilution, Novocastra Laboratories Ltd., Newcastle upon Tyne, UK), a bond polymer refine detection/polymeric horseradish peroxidase-linker antibody conjugate system (Leica Biosystems), and against p57 protein (Kip2)”. The valuation of *p57* staining was based on “the presence or absence of nuclear staining of cytotrophoblasts and villous stromal cells”. “Diffuse nuclear staining in cytotrophoblasts and villous stromal cells were considered as positive result (PHM or HA)”. “Absent or < 10% nuclear staining in the cytotrophoblasts and villous stromal cells was considered negative” (17).

### CISH

Samples of 4 µm thickness tissue were prepared from paraffin-embedded blocks in which chorionic elements had been confirmed with H&E slides and sent for *CISH*. All specimens underwent *CISH* with a probe to the chromosome 17(*HER 2*)gene(*ZytoDot HER-2/CEN-17*) and *CISH2*. *CISH* for *Chr 2* and *Chr 17* for PHM was defined as 3 green dots or 3 red dots in trophoblastic cells and stromal cells (triploid). *CISH* for *Chr 2* and *Chr 17* for CHM or HA change or trisomic pregnancy was defined as 2 red dots or 2 green dots in trophoblastic cells and villi stromal cells (diploid) (10, 18, 19).* p57, CISH2, *and* CISH17* tests were used respectively as complementary tests differentiating CHM, PHM, and HA.

**Table 1:** Morphology criteria for diagnosis of hydatidiform moles

**Morphology criteria**	**CHM**	**PHM**	**HA**
**T. Atypia**	Mild/moderate-severe	Absent/mild	Absent
**Cistern**	Present	Present	Absent
**MTP**	Mild/moderate-severe	Mild	Absent
**Lace like**	Present	Present	Absent
**Large-INC**	Absent/present	Present	Absent
**Scalloping**	Absent/mild	Mild/moderate-severe	Absent
**Small-INC**	Absent/present	Present	Present
**Fibrillary.C**	Absent	Absent/present	Present
HA: Non-molar hydropic abortion, PHM: Partial hydatidiform mole, CHM: Complete hydatidiform mole, T. Atypia: Trophoblast atypia, MTP: Multifocal trophoblast proliferation, Large-INC: Large trophoblast inclusions, Small-INC: Small trophoblast inclusions, fibrillary.C: Fibrillary collagen			

### Ethical Considerations

This study was approved by the Ethics Committee of Shiraz University of Medical Sciences, Shiraz, Iran (Code: IR.SUMS.MED.REC.1396.S184). The work process was completely anonymous, and the results were reported to the patients. All the study steps agreed with the Helsinki Declaration of 1964.

### Statistical Analysis

Frequency and relative frequency were used to describe quantitative variables; in addition, Chi-square and Fisher's exact tests were used to analyze the data; Cohen's Kappa coefficient was also used to measure intra-rater reliability for qualitative items; Kappa coefficient between 0.01–0.20, 0.21–0.40, 0.41–0.60, 0.61–0.80, and 0.81–1.00 were slight, fair, moderate, substantial, and perfect agreements, respectively (https://idostatistics.com/cohen-kappa-free-calculator/#risultati), sensitivity, specificity, and accuracy with 95% CI were also estimated. IBM SPSS v.22 and MedCal v.20.015 software tools at a statistical significance level < 0.05 were used for all tests. Post hoc power analysis was done to estimate the power of a test given an observed effect size at the end of the study (https://wiki.socr.umich.edu/index.php/SMHS_PowerSensitivitySpecificity).

## 3. Results

**Figure 2:**
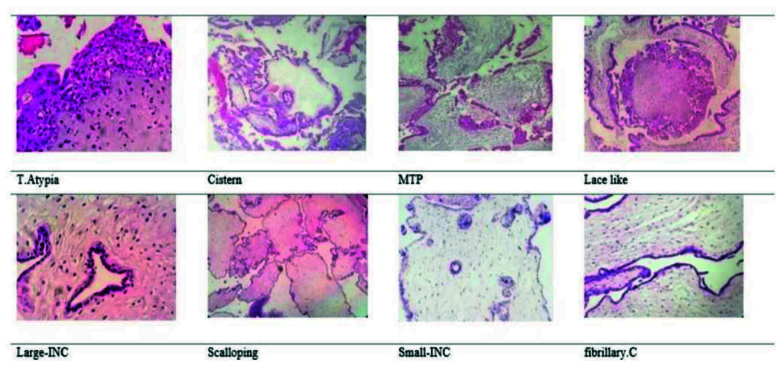
The morphologic criteria among 44 product of conception. T. Atypia: Trophoblast atypia, MTP: Multifocal trophoblast proliferation, Large-INC: Large trophoblast inclusions, Small-INC: Small trophoblast inclusions, Fibrillary.C: Fibrillary collagen.

Of 44 POCs, 70.5% (31/44), 22.7% (10/44), and 6.8% (3/44) of cases were diagnosed with PHM, CHM, and HA based on morphologic diagnosis, respectively; the morphologic criteria have been presented in figure 2. In addition, no significant difference was observed between PHM, CHM, and HA in terms of maternal age (p = 0.34) and gestational age (p = 0.42). The Kappa agreement coefficient between the 3 pathologists was estimated at 95% for PHMs. The morphological features of 44 cases by morphology, *CISH2, CISH17*, and *p57* diagnoses have been compared in table II.

**Figure 3:**
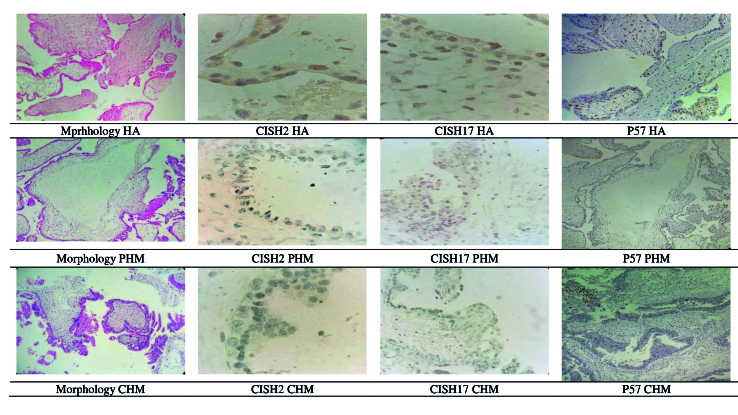
Diagnosed PHM, CHM, and HA by morphology, *CISH2, CISH17*, and *p57* among 44 product of conception. HA: Non-molar hydropic abortion, CISH: Chromogenic in situ hybridization,PHM: Partial hydatidiform mole, CHM: Complete hydatidiform mole.

In morphologic diagnosis, T. Atypia, cistern, MTP, lace-like, large INC, and fibrillary.C were significantly different among PHM, CHM, and HA groups; however, no significant differences were observed in scalloping and small-INC. In *CISH 2, CISH 17*, and *p57* diagnosis groups, MTP and small-INC were significantly different; however, T. Atypia, cistern, lace-like, large INC, fibrillary.C, and scalloping were not significantly different. Diagnosed PHM, CHM, and HA by morphology, *CISH2, CISH17*, and *p57* have been presented in figure 3.

18 out of 44 (40.9%) cases were diagnosed with CHM based on *p57*. The morphological features of the remaining 26 PHM cases by morphology, *CISH2*, and *CISH17* diagnosis have been compared in table III.

PHM and HA were 23 and 3 cases for *CISH2* and *CISH17*. In morphology, *CISH2* and *CISH17* diagnostic groups, cistern, MTP, lace-like, and fibrillary.C were significantly different among PHM and HA groups; however, T. Atypia, large INC, scalloping, and small INC did not significantly differ. The Kappa agreement coefficient between *CISH2* and *CISH17* has been shown in table IV.

There were perfect agreements between *CISH2* and *CISH17* when diagnosing the chromosomal ploidy of PHM (23), CHM (20), and HA (1); however, the agreements between morphology diagnosis and *CISH2* and morphology diagnosis and *CISH17* were moderate.

Post hoc power analysis of *CISH2* and *CISH17* discriminating between triploid and diploid in POCs given the estimated sensitivity and specificity has been presented in table V.

**Table 2:** Comparison of morphology features of 44 cases by morphology, *CISH2, CISH17, *and* p57* diagnosis

Morphology criteria		Morphology diagnosis			P-value	*CISH2* diagnosis		P-value	*CISH17* diagnosis		P-value	*p57* diagnosis		P-value
		**PHM**	**CHM**	**HA**		**PHM**	**CHM**		**PHM**	**CHM**		**PHM/HA**	**CHM**	
Total		31 (70.50)	10 (22.70)	3 (6.80)		24 (54.50)	20 (45.50)		23 (52.30)	21 (47.70)		26 (59)	18 (41)	
T. Atypia														
	**Absent**	3 (9.70)	0 (0)	2 (66.70)	0.02^*^	3 (12.50)	2 (10)	0.77^*^	3 (13)	2 (9.50)	0.80^*^	5 (19.20)	0 (0)	0.11^*^
	**Mild**	25 (80.60)	8 (80)	1 (33.30)		19 (79.20)	15 (75)		18 (78.30)	16 (76.20)		19 (73.10)	15 (83.30)	
	**Moderate-severe**	3 (9.70)	2 (20)	0 (0)		2 (8.30)	3 (15)		2 (8.70)	3 (14.30)		2 (7.70)	3 (16.70)	
Cistern														
	**Absent**	6 (19.40)	3 (30)	3 (100)	0.01^*^	7 (29.20)	5 (25)	0.51^†^	6 (26.10)	6 (28.60)	0.85^†^	9 (34.60)	3 (16.70)	0.18^*^
	**Present**	25 (80.60)	7 (70)	0 (0)		17 (70.80)	15 (75)		17 (73.90)	15 (71.40)		17 (65.40)	15 (83.30)	
MTP														
	**Absent**	0 (0)	0 (0)	2 (66.70)	< 0.001^*^	0 (0)	2 (10)	0.03^*^	0 (0)	2 (9.50)	0.01^*^	2 (7.70)	0 (0)	0.01^*^
	**Mild**	20 (64.50)	1 (10)	1 (33.30)		16 (66.70)	6 (30)		16 (69.60)	6 (28.60)		17 (65.40)	5 (27.80)	
	**Moderate-severe**	11 (35.50)	9 (90)	0 (0)		8 (33.30)	12 (60)		7 (30.40)	13 (61.90)		7 (26.90)	13 (72.20)	
Lace like														
	**Absent**	0 (0)	0 (0)	1 (33.30)	< 0.001^*^	0 (0)	1 (5)	0.45^*^	0 (0)	1 (4.80)	0.20^*^	1 (3.80)	0 (0)	0.40^*^
	**Present**	31 (100)	10 (100)	2 (66.70)		24 (100)	19 (95)		23 (100)	20 (95.20)		25 (96.20)	18 (100)	
Large-INC														
	**Absent**	8 (25.80)	8 (80)	2 (66.70)	< 0.001^*^	8 (33.30)	10 (50)	0.20^†^	7 (30.40)	11 (52.40)	0.13^†^	9 (34.60)	9 (50)	0.30^†^
	**Present**	23 (74.20)	2 (20)	1 (33.30)		16 (66.70)	10 (50)		16 (69.60)	10 (47.60)		17 (65.40)	9 (50)	
Scalloping														
	**Few**	17 (54.80)	7 (70)	2 (66.70)	0.90^*^	11 (45.80)	15 (75)	0.10^*^	11 (47.80)	15 (71.40)	0.22^*^	13 (50)	13 (72.20)	0.28^*^
	**Some**	13 (41.90)	3 (30)	1 (33.30)		12 (50)	5 (25)		11 (47.80)	6 (28.60)		12 (46.20)	5 (27.80)	
	**Many**	1 (3.20)	0 (0)	0 (0)		1 (4.20)	0 (0)		1 (4.30)	0 (0)		1 (3.80)	0 (0)	
Small-INC														
	**Absent**	5 (16.10)	2 (20)	1 (33.30)	0.75^*^	1 (4.20)	7 (35)	0.01^*^	1 (4.30)	7 (33.30)	0.01^*^	2 (7.70)	6 (33.30)	0.03^*^
	**Present**	26 (83.90)	8 (80)	2 (66.70)		23 (95.80)	13 (65)		22 (95.70)	14 (66.70)		24 (92.30)	12 (66.70)	
Fibrillary.C														
	**Absent**	29 (93.50)	10 (100)	0 (0)	< 0.001^*^	22 (91.70)	17 (43.60)	0.41^*^	21 (91.30)	18 (85.70)	0.56^*^	21 (80.80)	18 (100)	0.06^*^
	**Present**	2 (6.50)	0 (0)	3 (100)		2 (8.30)	3 (15)		2 (8.70)	3 (14.30)		5 (19.20)	0 (0)	
Data presented as n (%). *Fisher’s exact test, †Chi-square test, T. Atypia: Trophoblastic atypia, MTP: Multifocal trophoblast proliferation, Lace like: Lace like trophoblasts, Large-INC: Large trophoblastic inclusion, Small-INC: Small trophoblastic inclusion, Fibrillary.C: Fibrillary collagen, HA: Non-molar hydropic abortion, PHM: Partial hydatidiform mole, CHM: Complete hydatidiform mole														

**Table 3:** Comparison of morphological features of 26 PHM ancillary *p57* cases by morphological diagnosis*, CISH2* diagnosis, and *CISH17* diagnosis (n = 26/each)

Morphology criteria		Morphology diagnosis		P-value	*CISH2* diagnosis		P-value	*CISH17* diagnosis		P-value
		**PHM**	**HA**		**PHM**	**HA**		**PHM**	**HA**	
Total		23 (88.46)	3 (11.54)		23 (88.46)	3 (11.54)		23 (88.46)	3 (11.54)	
T. Atypia										
	**Absent**	3 (13)	2 (66.70)	0.08^*^	3 (13)	2 (66.70)	0.08^*^	3 (13)	2 (66.70)	0.08^*^
	**Mild**	18 (78.30)	1 (33.30)		18 (78.30)	1 (33.30)		18 (78.30)	1 (33.30)	
	**Moderate-severe**	2 (8.70)	0 (0)		2 (8.30)	0 (0)		2 (8.70)	0 (0)	
Cistern										
	**Absent**	6 (26.10)	3 (100)	0.01^*^	6 (21.60)	3 (100)	0.01^*^	6 (26.10)	3 (100)	0.01^*^
	**Present**	17 (73.90)	0 (0)		17 (73.90)	0 (0)		17 (73.90)	0 (0)	
MTP										
	**Absent**	0 (0)	2 (66.70)	< 0.001^*^	0 (0)	2 (66.70)	< 0.001^*^	0 (0)	2 (66.70)	< 0.001^*^
	**Mild**	16 (69.60)	1 (33.30)		16 (69.60)	1 (33.30)		16 (69.60)	1 (33.30)	
	**Moderate-severe**	7 (30.40)	0 (0)		7 (30.40)	0 (0)		7 (30.40)	0 (0)	
Lace like										
	**Absent**	0 (0)	1 (33.30)	< 0.001^*^	0 (0)	1 (33.30)	< 0.001^*^	0 (0)	1 (33.30)	< 0.001^*^
	**Present**	23 (100)	2 (66.70)		23 (100)	2 (66.70)		23 (100)	2 (66.70)	
Large-INC										
	**Absent**	7 (30.40)	2 (66.70)	0.21^*^	7 (30.40)	2 (66.70)	0.21^*^	7 (30.40)	2 (66.70)	0.21^*^
	**Present**	16 (69.60)	1 (33.30)		16 (69.60)	1 (33.30)		16 (69.60)	1 (33.30)	
Scalloping										
	**Few**	11 (47.80)	2 (66.70)	0.80^*^	11 (47.80)	2 (66.70)	0.81^*^	11 (47.80)	2 (66.70)	0.80^*^
	**Some**	11 (47.80)	1 (33.30)		11 (47.80)	1 (33.30)		11 (47.80)	1 (33.30)	
	**Many**	1 (4.30)	0 (0)		1 (4.30)	0 (0)		1 (4.30)	0 (0.0)	
Small-INC										
	**Absent**	1 (4.30)	1 (33.30)	0.07^*^	1 (4.30)	1 (33.30)	0.07^*^	1 (4.30)	1 (33.30)	0.07^*^
	**Present**	22 (95.70)	2 (66.70)		22 (95.70)	2 (66.70)		22 (95.70)	20 (66.70)	
Fibrillary.C										
	**Absent**	21 (91.30)	0 (0)	< 0.001^*^	21 (91.30)	0 (0)	< 0.001^*^	21 (91.30)	0 (0)	< 0.001^*^
	**Present**	2 (8.70)	3 (100)		2 (8.70)	3 (100)		2 (8.70)	3 (100)	
Data presented as n (%), T. Atypia: Trophoblastic atypia, MTP: Multifocal trophoblast proliferation, Lace like: Lace like trophoblasts, Large-INC: Large trophoblastic inclusion, Small-INC: Small trophoblastic inclusion, Fibrillary.C: Fibrillary collagen, HA: Non-molar hydropic abortion, PHM: Partial hydatidiform mole, CHM: Complete hydatidiform mole, *Fisher’s exact test										

**Table 4:** Kappa agreement coefficients between *CISH2* and *CISH17*, morphology and *CISH2*, and morphology and *CISH17*

Feature		*CISH17*		Feature		*CISH2*		Feature		*CISH17*	
		**Triploid (23)**	**Diploid (21)**			**Triploid (23)**	**Diploid (3)**			**Triploid (23)**	**Diploid (3)**
*CISH2*				**Morphology diagnosis**					**Morphology diagnosis**		
	**Triploid (24)**	23	1		**PHM (23)**	23	8		**PHM (23)**	23	8
	**Diploid (20)**	0	20		**CHM (3)**	1	9		**CHM (3)**	0	10
	Kappa = 95.40% SD = 0.04 p < 0.001				Kappa = 52% SD = 0.13 p < 0.001^*^				Kappa = 58% SD = 0.12 p < 0.001^*^		
	Accuracy = 95.26% (95% CI: 84.25-99.38) Sensitivity = 100% (95% CI: 85.18-100) Specificity = 95% (95% CI: 76.18-99.88)				Accuracy = 53.16% (95% CI: 36.96-68.9) Sensitivity = 96% (95% CI: 78.88-99.89) Specificity = 53% (95% CI: 27.81-77.02)				Accuracy = 55.79% (95% CI: 39.45-71.25) Sensitivity = 100% 95% CI: 85.18-100) Specificity = 55% (95% CI: 30.76-78.48)		
Triploid: Partial hydatidiform mole, Diploid: Complete hydatidiform mole or hydropic abortion, PHM: Partial hydatidiform mole, CHM: Complete hydatidiform mole,											

**Table 5:** Post hoc power analysis of *CISH2* and *CISH17* discriminating between triploid and diploid in product of specimens

Feature		*CISH17*	
		**Triploid (23)**	**Diploid (21)**
*CISH2*	**Triploid (24)**	23 (true positive = 100%)	1 (false positive = type error = 0.05)
	**Diploid (20)**	0 (false negative = type II error = 0)	20 (true negative = 95%)
Test interpretation	Power^*^ = $\;1-\frac{false\;negative}{false\;negative+true\;positive}=100\%$		
*Power = 1-type II error, the power of CISH2 and CISH17 tests discriminating between triploid and diploid in POC specimens was estimated at 100%. CISH: Chromogenic in situ hybridization			

## 4. Discussion

From 44 POCs, 31, 10, and 3 cases were diagnosed as PHM, CHM, and HA based on the morphology diagnosis by 3 pathologists with a 95% agreement for PHM. Moreover, there was perfect agreement between *CISH2* and *CISH17*, differentiating triploidy and diploidy. It is easy to diagnose CHM by morphology unless it is in lower gestational age or early CHM which is confused with HA. Based on the current findings, it is better to evaluate and diagnose early CHM by doing *p57* before sending samples suspected of PHM for *CISH* diagnosis. To increase the accuracy of the diagnosis, it is suggested to perform the test with 2 chromosomes (2 and 17). Due to the high agreement coefficient between *CISH2* and *CISH17*, lower prevalence of trisomy of chromosome 17 in POCs, and *CISH17* more availability in laboratories, it is more cost-effective to do only *CISH17*. Further studies are suggested to compare the *CISH* method's accuracy with other ploidy studies, such as flow cytometry karyotyping and *FISH*.

In agreement with the current result, it was reported that morphology by itself cannot result in an accurate diagnosis of PHM; they also showed that a genotype study is also necessary to differentiate between PHM and CHM (20).

A study done on 89 POCs containing 54 PHM and 35 HA showed that fibrillary.C was more common in HA, while T. Atypia, MTP, and cistern were more common in PHM. These findings were in line with the current results. Also, they presented an older maternal age in HA in comparison with PHM (average age of 37.2 vs. 29.5 yr); however, no difference was observed between groups regarding gestational age (10). Moreover, in our study, no significant differences were observed between groups in terms of maternal age and gestational age.

In a study performed on 45 POCs consisting of 36 CHM and 9 PHM, classified according to the morphology criteria, including T. Atypia, cistern, large-INC, small-INC, and lace-like fibrillary.C, no significant differences were observed between groups in terms of gestational age and maternal age, which is in line with our study (21).

It was revealed that flow cytometry karyotyping for detecting the presence or absence of maternal genome content was the gold standard, also *CISH* for DNA ploidy assessment was used. The pericentromeric area of chromosomes 11, 16, X, and Y were labeled by biotin-1. A pair of 6 primers on 4 different chromosomes were tested by genotyping 34/45 cases including 31/36 CHM and 3/9 PHM that resulted in no maternal genome content which was genetically introduced as CHM. About 11/45 cases including 5/36 CHM and 6/9 PHM, presented maternal genome content, which was genetically introduced as PHM. In the cited study, morphologic diagnosis and the genetic result showed a correlation of 88% (37/45 cases). This technique was compared with *CISH*, and it was concluded that all HMs with maternal genome content were presented with a triploid pattern and all HMs without maternal content were presented with a diploid pattern (21). Their findings strengthen the accuracy of the *CISH* technique for differentiating CHM from PHM, which is in line with the current work.

In comparison with the histology and genetic study, they concluded that morphology criteria were more reliable for diagnosis of CHM than PHM (91% or 31/34 than 55% or 6/11, p < 0.001). Our results also showed that morphology was more reliable for CHM diagnosis than PHM. In a comparison of the *CISH* and genetic study, a good agreement between the 2 techniques for the diagnosis of the hydatidiform mole was seen, especially in the cases with challenging morphologic pictures (21).

A study conducted on 22 POCs included 6 CHM, 10 PHM, and 6 HA, after histological evaluation according to the morphologic criteria, they used the *CISH* technique with a *chromosome 17 probe *(*HER 2/CEN*), dual-colored (red and blue), on all specimens. In our study, morphologic criteria and DNA ploidy techniques were the same. Their results of counting red and blue signals in the trophoblastic cells and stromal cells were as follows: 9/10 (90%) of histologically diagnosed PHM showed a *HER 2* signal value of 2.92 (triploid) and 1/10 PHM had a *HER 2* signal value of 2.5. Decidua tissue was selected as an internal control. A total of 11/12 remaining cases (91.7%) presented a *HER 2* signal value of 2.06 and 1/12 histologically diagnosed hydropic changes presented a *HER 2* signal value of 2.35. Hence, they concluded that *CISH chromosome 17* to differentiate diploid from triploid had a sensitivity of 90%, a specificity of 91.6%, and an agreement of 90.9%, which was in line with our study. Also similar to our study, no significant difference was observed between the patients in terms of maternal or gestational age (9).

In a study on 51 POCs, including 18 CHMs, 24 PHMs, and 9 HAs based on morphology characters, they performed a diagnostic algorithm for the diagnosis of HM according to *p57 *immunohistochemistry and a DNA ploidy *FISH* study. All 18 (100%) cases with morphology diagnosed as CHMs demonstrated a diploid genotype. Among 24 cases with PHMs, only 9 (37.5%) cases had a triploid genotype, and the remaining (62.5%) had a diploid genotype. All cases with HA (100%) displayed a diploid genotype. In this study, after a combination of *p57* immunohistochemistry, DNA ploidy *FISH* study, and morphology in 51 cases, the diagnostic results were as follows: 27 CHMs, 9 PHMs, and 15 HAs. The results showed that the diagnostic accuracy based on morphology alone in the diagnosis of CHM and PHM was 78.4% and 70.6%, respectively. It can lead to a misdiagnosis, so the use of other diagnostic methods such as *FISH* and *CISH* is recommended in this study, empowering the current study's aim (22).

### Strength and limitation

The utmost effort and precision have been made in defining and subtracting the variables and outcomes; all steps have been carefully monitored and checked to minimize possible bias and enhance the validity of the results. As the other strong point of the work, 3 pathologists got involved in the morphology assessment of the PHM, CHM, and HA to minimize the error. Although not all POCs were included in the study and we faced attrition of POCs, the sample size was large enough to detect the differences compared with the previous works. A limitation of the work was the retrospective design, which resulted in the unavailability of fresh specimens to conduct the gold standard methods like *FISH* and flow cytometry karyotyping. These supplementary tests could compare the results and improve the accuracy of the diagnosis.

## 5. Conclusion

Based on the current finding, *CISH2* and *CISH17* enjoyed perfect agreement in diagnosing HMs; in addition, their absolute power discriminating between triploid and diploid revealed that they could be used as surrogate markers for ploidy. From 44 POCs, 31, 10, and 3 cases were diagnosed as PHM, CHM, and HA based on the morphology diagnosis. It is easy to diagnose CHM by morphology unless it is in lower gestational age or early CHM which is confused with HA. Based on the current findings, it is better to evaluate and diagnose early CHM by doing *p57* before sending samples suspected of PHM for *CISH* diagnosis; although, to increase the accuracy of the diagnosis, it is suggested to perform the test with 2 *chromosomes *(2 and 17). Due to the high agreement coefficient between *CISH2* and *CISH17*, lower prevalence of trisomy of chromosome 17 in POCs, no need for fresh specimens of POCs, and *CISH17* availability in laboratories, it is more cost-effective to perform only *CISH17*. Prospective studies on fresh specimens are suggested comparing the *CISH* method's accuracy with other ploidy studies, such as flow cytometry karyotyping and *FISH*. Some advantages and disadvantages of *CISH* compared with flow cytometry karyotyping and *FISH* are as follows:

• *CISH* has a quick turnaround time as *FISH* providing the ability to perform shorter DNA ploidy in urgent cases.

• In POCs, *CISH* enjoys high true positive and high true negative values in discriminating between triploid and diploid; however, “the relatively high false positive and false negative rates of *FISH* technique complicates its application for analyzing small population of pathological cells” (23).

• *CISH* correlates with *HER2* status and morphological features; however, FISH is an immunofluorescence staining technique needing immunofluorescence technique, which makes it more costly and less available in pathologic laboratories.

• Flow cytometry karyotyping provides a comprehensive view of the genome, while CISH can detect cryptic or submicroscopic genetic abnormalities and identify genetic abnormalities in nondividing cells. In addition, flow cytometry karyotyping needs more time and is more costly than *CISH*.

##  Data Availability

Data is available at corresponding author contact (marjan.zare@gmail.com).

##  Author Contributions

M. Akbarzadeh-Jahromi contributed to the conception, design, conduction, supervision, and drafting the manuscript. T. Taheri, F. Sari Aslani, A. Safaei, and F. Pouraminaee contributed to the conception, design, conduction, collection of the data, and drafting the manuscript. M. Zare contributed to the design, analysis of the data, interpretation of the results, drafting the manuscript, and reviewing the work critically. All authors approved the final manuscript and take responsibility for the integrity of the data.

##  Conflict of Interest

The authors declare that there is no conflict of interest.
